# Supplementary material for: Social environment-based opportunity costs dictate when people leave social interactions
Source: Commun Psychol. 2024 May 9;2:42. doi: 10.1038/s44271-024-00094-5 (PMC11081926; doi:10.1038/s44271-024-00094-5)
Supplement: Supplementary file 1 — Supplementary Materials [file 44271_2024_94_MOESM1_ESM.pdf]

Supplementary materials

Supplementary methods

Experimental paradigm

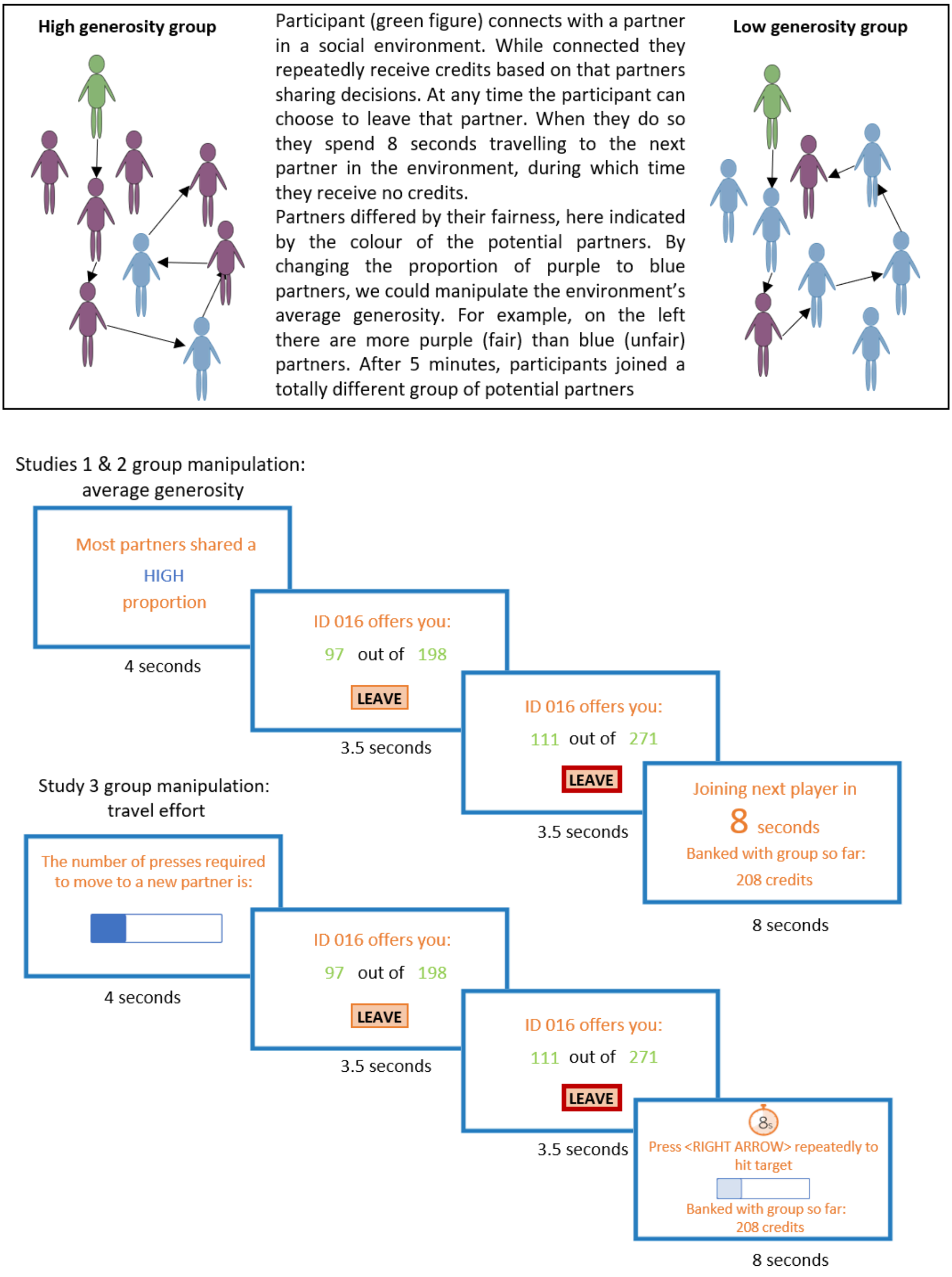

Supplementary Figure 1: Top: Diagrammatic representation of the task as explained to participants. Bottom: Task presentation for Studies 1 – 3. This differs to the presentation in Study 4, which can be seen in the main text (Figure 1). For

Studies 1 and 2 the environment manipulation was average generosity. For Study 3 it was effort required to move between partners

Supplementary Table 1: Demographics by study, after exclusions.

	Study 1	Study 2	Study 3	Study 4
N	19	25	24	81
%F	76.4	65.4	73.1	49.4
Age	27.6 (SD 5.3)	23.7 (SD 4.5)	24.2 (SD 5.0)	30.7 (SD 8.5)
Environment manipulation	Generosity	Generosity	Effort	Generosity

Sample size justification

We ran the statistical model on 5000 simulated datasets based on the fixed effects and variance of the random effects from the analysis of Study 1. Power for each fixed effect was calculated as the proportion of simulations where $p < 0.05$. This analysis suggested data from 25 participants would give us over 90% power to detect main effects of partner and environment type, and over 80% power to detect an interaction, with alpha at 0.05. Therefore, we set this as our target sample size for Studies 2 and 3. We were uncertain as to a likely effect size for the exploratory analyses examining the role of depressive symptoms in Study 4, so aimed to recruit 100 participants for this study. It is recommended that post-hoc power analyses are not used as an estimate of power (Dziak et al., 2020). However, a recommended alternative is to calculate the bootstrapped confidence interval of the beta value and if 0 is not within the interval it provides evidence that the study has sufficient power. We recognise that sufficient power for three-way interactions is difficult to achieve, so this procedure has been carried out on the model parameters from the depression analysis of Study 4 (Supplementary Figure 2).

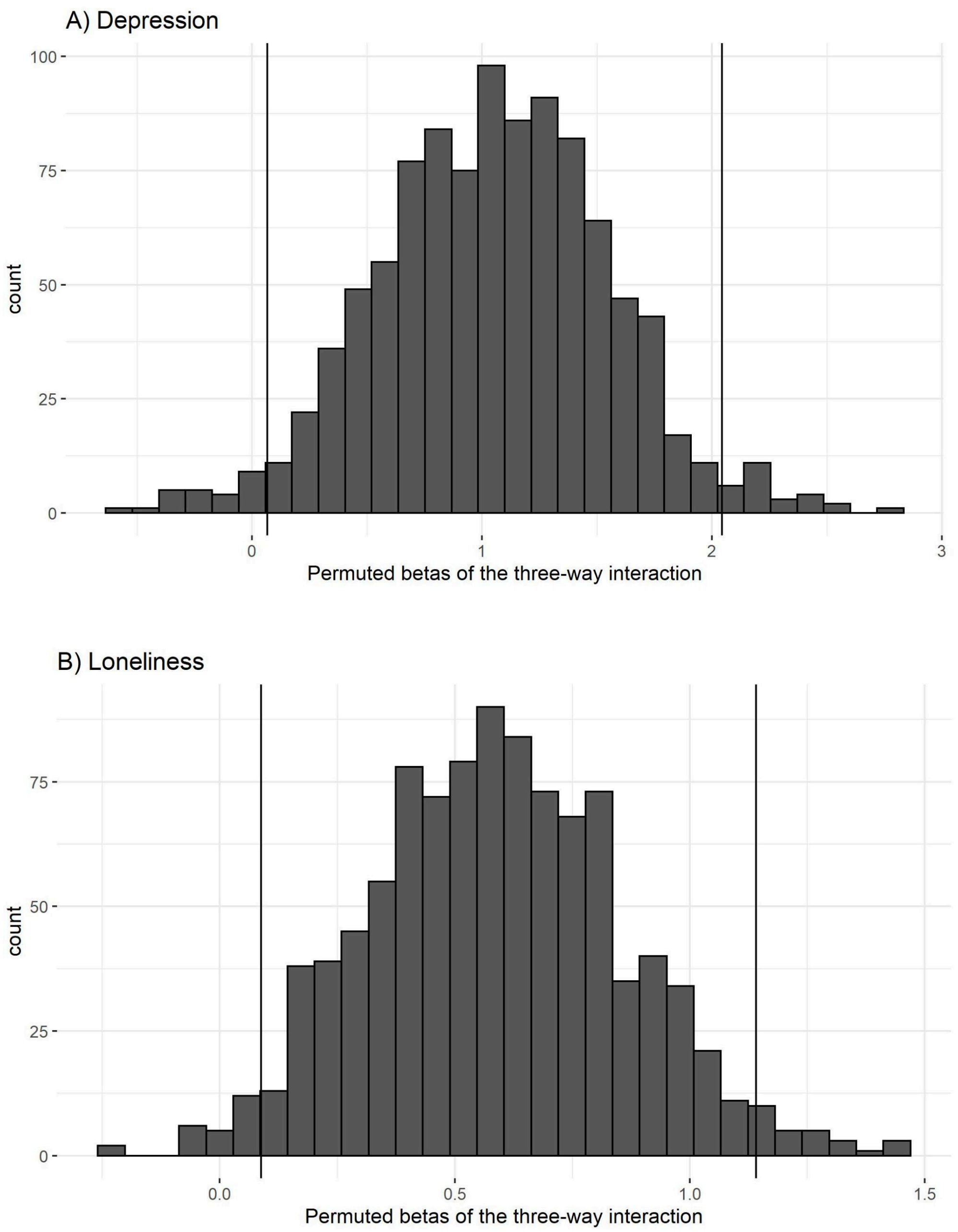

Supplementary Figure 2: Histogram of 1000 bootstrapped beta values for the three-way interaction in the depression (A) and loneliness (B) analyses of Study 4. Vertical lines indicate the bootstrapped 95% confidence intervals. The exclusion of zero from these intervals suggests sufficient power to detect this effect.

Supplementary results

Behavioural analyses

Control analysis (Study 1 & 2) – excluding zero fairness trials

The fairness trajectories used in Study 1 and Study 2 (see methods for details) allowed for fairness to drop to zero. Continuing to interact with a partner while they share no money is a legitimate behaviour in the task, but it could also be a sign of inattention. We repeated the analyses from both study 1 and study 2, excluding decisions made after the fairness had dropped to zero. The main effects of partner and environment type remained statistically significant for both studies, while the interaction between predictors was non-significant for both studies (Supplementary Table 1).

Supplementary Table 2: Results from linear mixed models for Study 1 & Study 2 when excluding decisions when fairness was equal to zero

Study	Predictor	*b*	SE	95% CI	*t*	*p-value*	X^2	*p-value*
1	Partner	-8.21	0.77	-9.73, -6.69	-10.619	< 0.001	113.36	< 0.001
	Environment	2.53	0.78	1, 4.07	3.237	0.001	11.01	0.001
	Partner*environment interaction	-2.00	1.54	-5.03, 1.03	-1.297	0.195	1.68	0.195
2	Partner	-10.15	1.21	-12.65, -7.65	-8.400	< 0.001	70.98	< 0.001
	Environment	2.52	1.13	0.08, 4.95	2.221	0.044	4.87	0.027
	Partner*environment interaction	1.03	1.56	-2.04, 4.10	0.658	0.51	0.43	0.510

Supplementary analysis – fairness at leaving

We examined whether the fairness at the time of choosing to leave a partner differed by partner type or social environment for each study. Across all studies there was a main effect of both partner type and social environment, and no interaction. In all cases, the fairness at leaving was lower for less fair partners than fairer partners, and in low quality environments compared to high.

Supplementary Table 3: Results from linear mixed models for all studies, with fairness as the outcome variable

Study	Predictor	*b*	SE	95% CI	*t*	*p-value*	X^2	*p-value*
1	Partner	-2.63	0.63	-3.97, -1.29	-4.146	0.001	16.82	< 0.001
	Environment	-2.08	0.90	-3.69, -0.20	-2.316	0.032	5.48	0.019
	Partner*environment interaction	1.46	0.94	-0.38, 3.30	1.56	0.120	2.42	0.120
2	Partner	-3.15	0.75	-4.71, -1.59	-4.170	< 0.001	17.40	< 0.001
	Environment	-2.68	0.92	-4.58, -0.78	2.914	0.008	8.51	0.004
	Partner*environment interaction	-0.58	0.99	-2.53, 1.36	-0.588	0.557	0.35	0.557
3	Partner	-1.99	0.37	-2.73, -1.25	-5.311	< 0.001	27.95	< 0.001
	Environment	-1.23	0.38	-1.96, -0.49	-3.268	0.001	10.48	0.001
	Partner*environment interaction	-0.40	0.75	-1.87, 1.07	-0.537	0.591	0.29	0.591
4	Decay rate (partner type)	0.02	0.001	0.01, 0.02	10.877	< 0.001	120.33	< 0.001
	Environment	-0.01	0.004	-0.02, 0.00	-2.142	0.035	4.54	0.033
	Partner*environment interaction	-0.004	0.002	-0.1, 0.00	-1.724	0.085	2.97	0.085

Supplementary analysis – earnings

We examined whether the amount earned per partner differed by partner type or social environment for each study. Across all studies there was a main effect of both partner type and social environment. In all cases, participants earned less with unfair partners than fairer partners, and earned more in low quality environments than high quality environments. Since participants accumulate reward while interacting with a partner, and earn no reward while travelling between partners, this pattern of results is to be expected. Leaving times were earlier in a high opportunity cost environment, meaning more time was spent travelling, and therefore less reward earned. People spent more time interacting with fair partners than unfair partners, and therefore earned more money with those partners. There was a partner by environment interaction in Study 1, but this did not replicate across studies.

Supplementary Table 4: Results from linear mixed models for all studies, with earnings as the outcome variable

Study	Predictor	*b*	SE	95% CI	*t*	*p-value*	X^2	*p-value*
1	Partner	-343.28	40.68	-428.74, -257.82	-8.44	0.001	71.84	< 0.001
	Environment	72.42	32.11	5.06, 139.77	2.255	0.037	5.23	0.022
	Partner*environment interaction	-71.00	29.94	-129, -12.23	-2.371	0.018	5.62	0.018
2	Partner	-331.19	36.38	-406.27, -256.11	-9.103	< 0.001	82.86	< 0.001
	Environment	78.98	29.58	17.88, 140.99	2.670	0.14	7.11	0.008
	Partner*environment interaction	-13.99	30.87	-74.56, 46.58	-0.453	0.651	0.21	0.650
3	Partner	-214.50	33.59	-280.44, -148.56	-6.385	< 0.001	41.51	< 0.001
	Environment	145.77	33.63	79.75, 211.78	4.334	< 0.011	18.50	< 0.001
	Partner*environment interaction	40.72	67.05	-90.90, 172.34	0.607	0.544	0.37	0.544
4	Decay rate (partner type)	758.15	60.79	637.05, 879.25	12.472	< 0.001	156.44	< 0.001
	Environment	324.72	106.27	112.26, 537.18	3.056	0.003	9.38	0.002
	Partner*environment interaction	-51.24	55.59	-160.22, 57.74	-0.922	0.357	0.85	0.357

Supplementary analysis – do depression/loneliness scores predict earnings?

To test whether the altered behaviour seen in participants with higher self-reported depression and loneliness was more economically rational, we carried out two regression analyses predicting overall earnings by depression or loneliness. Supplementary Table 5 shows that neither of these predictors were significant.

Supplementary Table 5: Results from regression analyses testing the relationship between overall task earnings and depression/loneliness

Predictor	*b*	SE	95%CIs	*t*	*p-value*
Depression	1069	3307	-5566.3, 7705.15	77.229	0.749
Loneliness	225.53	1805.9	-3373.55, 3824.61	0.125	0.901

Drift diffusion model results

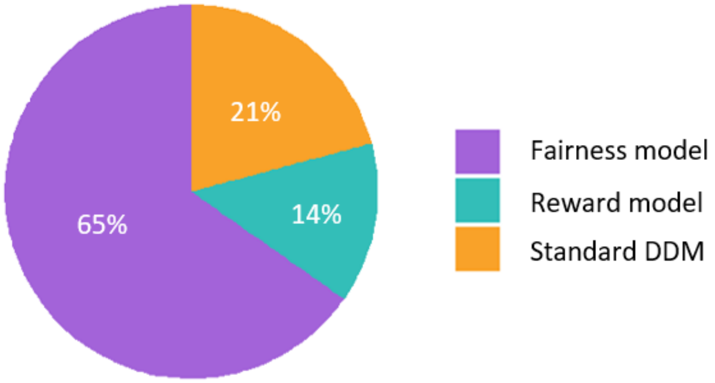

Supplementary Figure 3: Percentage of participants with each model as their winning model. The fairness adapted DDM was the best fitting model in 65% of participants

Mixed effect models on the non-winning modelled-simulated data

To further demonstrate that the reward model and standard DDM did not capture the shape of the experimental data we ran the statistical model on the simulated dataset from these models. Supplementary Table 4 shows how neither model captures both the partner-type and social environment effect present in the real data.

Supplementary Table 6: mixed model results for simulated data from non-winning DDMs

Model	Predictor	*b*	SE	95%CIs	*t*	*p-value*	X^2	*p-value*
Reward	Partner	0.002	.014	-0.28, 0.28	0.012	0.990	0.46	0.495
	Environment	-3.07	0.14	-3.35, -2.80	-21.705	<0.001	975.86	<0.001
	Partner*environment interaction	-0.1	0.20	-0.53, 0.25	-0.694	0.488	0.48	0.488
Standard DDM	Partner	0.05	0.14	-0.22, 0.32	0.383	0.702	1.09	0.297
	Environment	-0.07	0.14	-0.35, 0.19	-0.575	0.565	0.10	0.751
	Partner*environment interaction	0.10	.019	-0.28, 0.48	0.496	0.620	0.25	0.620

Fitted parameter values from winning DDM

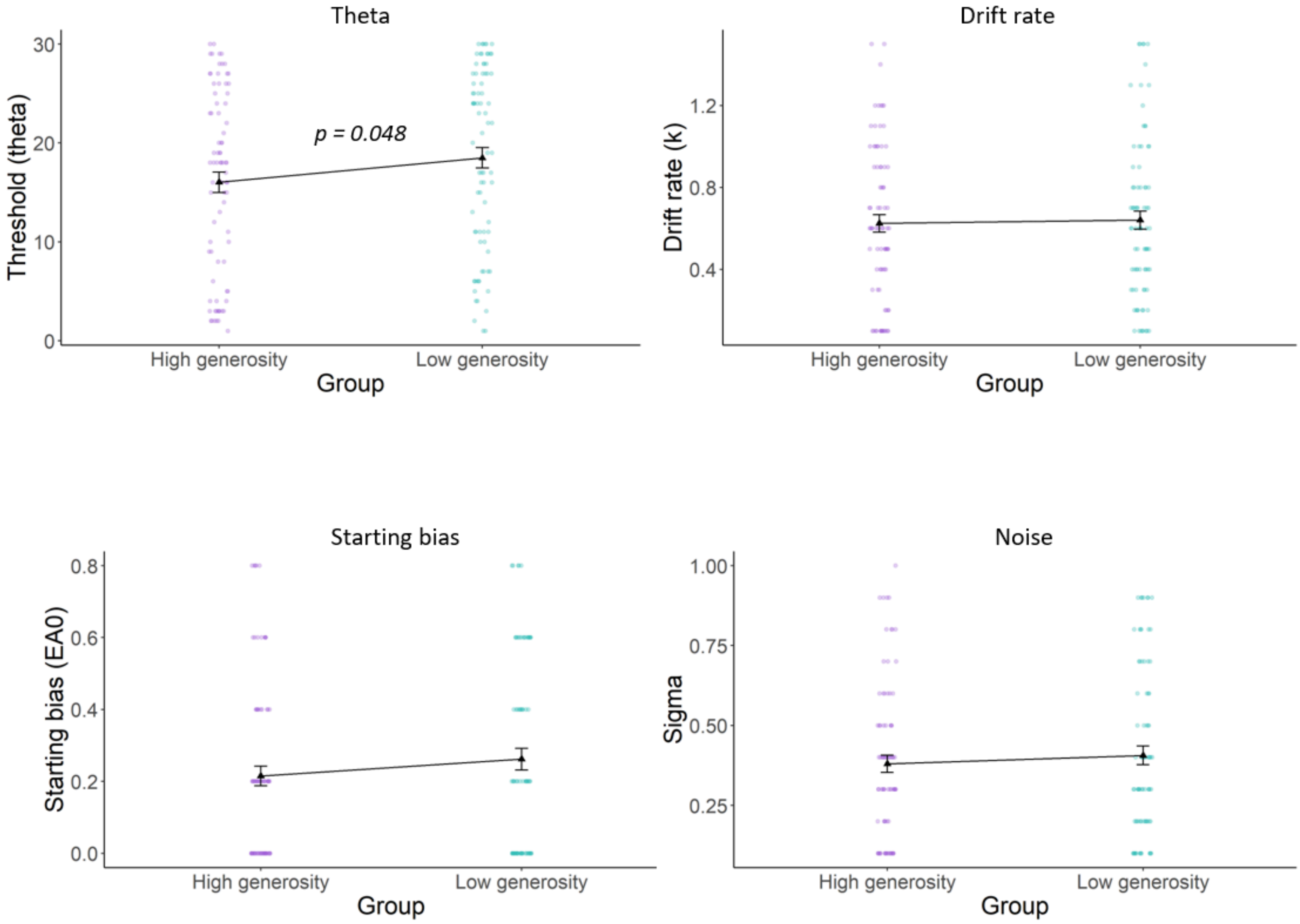

Supplementary Figure 4: fitted parameter values for the winning Fairness DDM. The threshold parameter, theta differed across environments. All other parameters did not differ significantly between social environments

Raw depression and loneliness scores

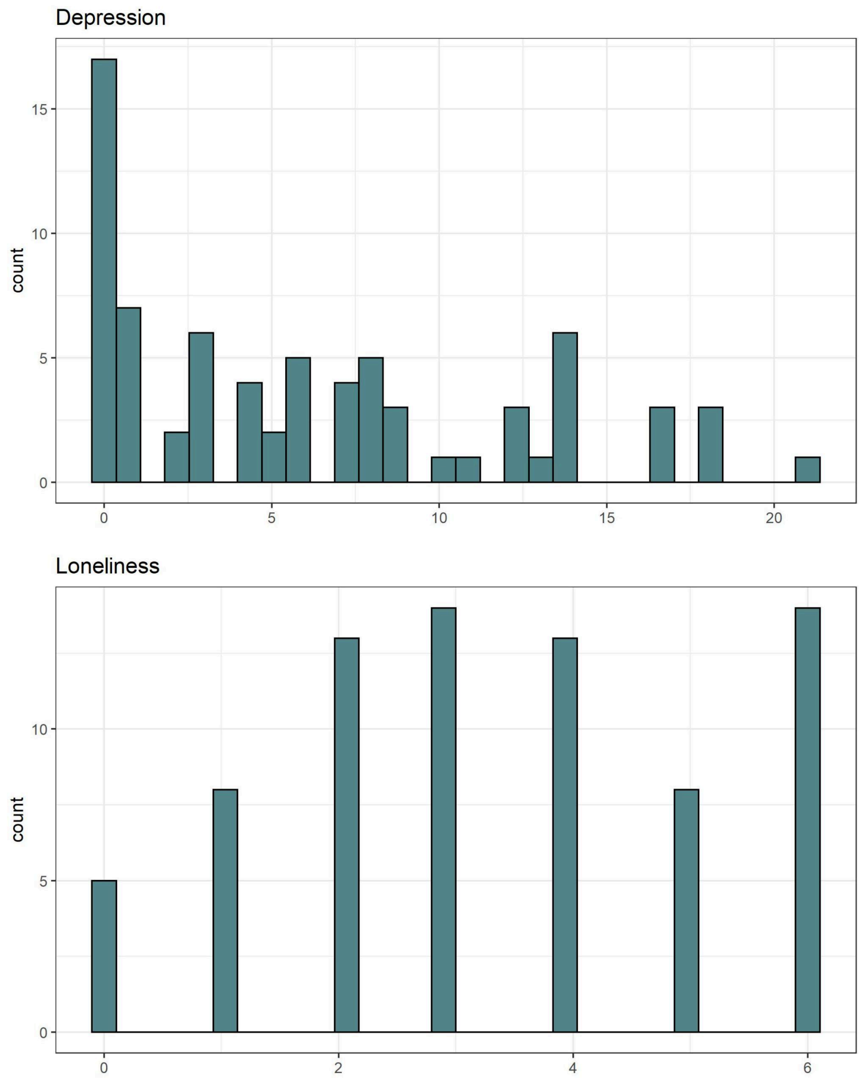

Supplementary Figure 5: Histograms of raw depression and loneliness scares, as measured by the Depression Anxiety and Stress Scales (DASS-21)(Henry & Crawford, 2005) and the De Long Gierveld Loneliness Scale (DGLS)(De Jong Gierveld & Van Tilburg, 2010)
