# Supplementary material for: Social environment-based opportunity costs dictate when people leave social interactions
Source: Commun Psychol. 2024 May 9;2:42. doi: 10.1038/s44271-024-00094-5 (PMC11081926; doi:10.1038/s44271-024-00094-5)
Supplement: Supplementary file 3 — Reporting Summary [file 44271_2024_94_MOESM3_ESM.pdf]

nature portfolio

Corresponding author(s): Anthony Gabay

Last updated by author(s): Sep 10, 2023

Reporting Summary

Nature Portfolio wishes to improve the reproducibility of the work that we publish. This form provides structure for consistency and transparency in reporting. For further information on Nature Portfolio policies, see our Editorial Policies and the Editorial Policy Checklist.

Statistics

For all statistical analyses, confirm that the following items are present in the figure legend, table legend, main text, or Methods section.

n/a	Confirmed	
☐	☒	The exact sample size (*n*) for each experimental group/condition, given as a discrete number and unit of measurement
☐	☒	A statement on whether measurements were taken from distinct samples or whether the same sample was measured repeatedly
☐	☒	The statistical test(s) used AND whether they are one- or two-sided<br>*Only common tests should be described solely by name; describe more complex techniques in the Methods section.*
☐	☒	A description of all covariates tested
☐	☒	A description of any assumptions or corrections, such as tests of normality and adjustment for multiple comparisons
☐	☒	A full description of the statistical parameters including central tendency (e.g. means) or other basic estimates (e.g. regression coefficient) AND variation (e.g. standard deviation) or associated estimates of uncertainty (e.g. confidence intervals)
☐	☒	For null hypothesis testing, the test statistic (e.g. *F*, *t*, *r*) with confidence intervals, effect sizes, degrees of freedom and *P* value noted<br>*Give P values as exact values whenever suitable.*
☒	☐	For Bayesian analysis, information on the choice of priors and Markov chain Monte Carlo settings
☐	☒	For hierarchical and complex designs, identification of the appropriate level for tests and full reporting of outcomes
☐	☒	Estimates of effect sizes (e.g. Cohen's *d*, Pearson's *r*), indicating how they were calculated

Our web collection on statistics for biologists contains articles on many of the points above.

Software and code

Policy information about availability of computer code

Data collection	The task was coded in PsychoPy version 2020.1.3(Peirce et al., 2019), and implemented through a Windows 10 PC (Studies 1 – 3) or hosted on Pavlovia (pavlovia.org) (Study 4).
Data analysis	All statistical analyses were carried out using R version 4.0.2(R Development Core Team, 2020). All models were analysed as linear mixed-effects models using the packages lme4, lmerTest, and car, with post-hoc analyses carried out with emmeans(Bates et al., 2015; Kuznetsova et al., 2017; Lenth, 2020). Data were visualised using the package ggplot2 and model visualisations used the package interactions (Long, 2019; Wickham, 2016).

For manuscripts utilizing custom algorithms or software that are central to the research but not yet described in published literature, software must be made available to editors and reviewers. We strongly encourage code deposition in a community repository (e.g. GitHub). See the Nature Portfolio guidelines for submitting code & software for further information.

Data

Policy information about availability of data

All manuscripts must include a data availability statement. This statement should provide the following information, where applicable:

- Accession codes, unique identifiers, or web links for publicly available datasets
- A description of any restrictions on data availability
- For clinical datasets or third party data, please ensure that the statement adheres to our policy

The task materials and data for this publication are accessible on the Open Science Foundation https://osf.io/urjen.

Human research participants

Policy information about studies involving human research participants and Sex and Gender in Research.

Reporting on sex and gender	Data was not collected on the basis of sex or gender, but self-reported sex (percent female) is reported in the manuscript. Analyses were not stratified by sex or gender as this was not relevant to the hypotheses being tested
Population characteristics	See below
Recruitment	See Research Sample below.
Ethics oversight	All studies were approved by The University of Oxford Central Research Committee (Studies 1 – 3 reference number R6061/RE001; Study 4 reference number R59122/RE001)

Note that full information on the approval of the study protocol must also be provided in the manuscript.

Field-specific reporting

Please select the one below that is the best fit for your research. If you are not sure, read the appropriate sections before making your selection.

☐ Life sciences ☒ Behavioural & social sciences ☐ Ecological, evolutionary & environmental sciences

For a reference copy of the document with all sections, see nature.com/documents/nr-reporting-summary-flat.pdf

Behavioural & social sciences study design

All studies must disclose on these points even when the disclosure is negative.

Study description	There a 4 studies reported in this manuscript, all with quantitative data
Research sample	175 participants (mean age = 27.6 (SD 7.6), range 18 – 51; 62% F). In studies 1 – 3, participants were recruited through an Oxford University participant database. In Study 4, participants were recruited through the online platform Prolific (www.prolific.co).
Sampling strategy	We ran the statistical model on 5000 simulated datasets based on the fixed effects and variance of the random effects from the analysis of Study 1. Power for each fixed effect was calculated as the proportion of simulations where $p < 0.05$. This analysis suggested data from 25 participants would give us over 90% power to detect main effects of partner and environment type, and over 80% power to detect an interaction, with alpha at 0.05. Therefore, we set this as our target sample size for Studies 2 and 3. We were uncertain as to a likely effect size for the exploratory analyses examining the role of depressive symptoms in Study 4, so aimed to recruit 100 participants for this study.
Data collection	In Studies 1 – 3, participants attended in-person. In these cases, the researcher sat with the participants while they viewed tasks instructions and were available to provide further explanations or to clarify points of the experiment. After practice runs of the task, the experimenter left the room, leaving the participant to complete the tasks alone.<br>In study 4, data was collected on Prolific (prolific.co). In these cases, there was no researcher present, and the participants' environments were beyond the control of the research team.
Timing	Data for Studies 1-3 were collected between March 2019 and December 2019. Study 4 data was collected in June 2020.
Data exclusions	Therefore, in Studies 1 – 3, we implemented an exclusion criterion that participants must have made an equal number of active decisions as there were environments. While this may remove participants from the analysis who legitimately chose to never leave a partner, it is a minimal criterion in order to ensure some engagement in the task (Study 1 excluded n = 1; Study 3 excluded n = 1). Study 4 included attention checks. Participants were excluded if they failed more than 25% of attention checks (n = 21). For all studies, we excluded trials where the leaving time was more than 2.5 standard deviations of the mean, on a within-participant, within-condition basis. Again, this was to protect against lapses in attention. These exclusion criteria were pre-established.

Non-participation	No participants dropped out/declined to participate
Randomization	Participants were not separated into experimental groups.

Reporting for specific materials, systems and methods

We require information from authors about some types of materials, experimental systems and methods used in many studies. Here, indicate whether each material, system or method listed is relevant to your study. If you are not sure if a list item applies to your research, read the appropriate section before selecting a response.

Materials & experimental systems

n/a	Involved in the study
☒	☐ Antibodies
☒	☐ Eukaryotic cell lines
☒	☐ Palaeontology and archaeology
☒	☐ Animals and other organisms
☒	☐ Clinical data
☒	☐ Dual use research of concern

Methods

n/a	Involved in the study
☒	☐ ChIP-seq
☒	☐ Flow cytometry
☒	☐ MRI-based neuroimaging
